# UNDERSTANDING AGING DIVERSITY THROUGH HUMANIST VALUES OF AGING: LOVE, HOPE, PEACE, AND STEWARDSHIP

**DOI:** 10.1093/geroni/igac059.038

**Published:** 2022-12-20

**Authors:** Stephen Fogle

**Affiliations:** University of Nebraska at Omaha, Omaha, Nebraska, United States

## Abstract

Humanist values of aging are relevant for understanding diversity in gerontological education because they are manifested across all cultures and ages. This paper proposes attention to four humanist values of aging: Love, Hope, Peace, Stewardship. This paper seeks opportunities for students to understand aging diversity in half century context: from traditional frames of race and sexuality to global conversations on language and geography. Drawing from stories about older adults this paper gathers diverse opportunities for understanding of humanist values of aging. Selections strive toward expanding representation of aging diversity and accessibility for students. Teaching with stories of love, hope, peace, and stewardship as humanist values of aging matches AGHE initiatives for incorporating humanities into gerontological education and opens opportunities for recognizing diversity representation in our society for all ages. Understanding aging diversity through stories of older adults engaged with humanist values of aging promises enriching educational experiences for lifelong learning.

